# Joint Symptoms, Aromatase Inhibitor-Related Adverse Reactions, Are Indirectly Associated with Decreased Serum Estradiol

**DOI:** 10.1155/2011/951260

**Published:** 2011-05-26

**Authors:** Junko Honda, Miyuki Kanematsu, Misako Nakagawa, Masako Takahashi, Taeko Nagao, Akira Tangoku, Mitsunori Sasa

**Affiliations:** ^1^Department of Surgery, Higashitokushima National Hospital, 1-1 Ohmukai-kita, Ootera, Itano, Tokushima 779-0193, Japan; ^2^Department of Oncological and Regenerative Surgery, Institute of Health Biosciences, The University of Tokushima, 3-18-15, Kuramoto-Cho, Tokushima 770-8509, Japan; ^3^Department of Surgery, Tokushima Breast Care Clinic, 4-7-7 Nakashimada-Cho, Tokushima 70-0052, Japan

## Abstract

*Background*. Joint symptoms (JSs) are problematic adverse drug reactions (ADRs) of aromatase inhibitors (AIs). Involvement of decreased serum estradiol (SE) has been suggested. *Patients and Methods*. 104 postmenopausal breast cancer patients administered an AI were prospectively investigated regarding various clinical parameters, JS and hot flashes as ADRs, and the SE level. *Results*. JS manifested in 31.7% of patients and hot flashes in 18.3%. Chi-square testing showed a significantly higher incidence of JS in several patient strata: <55 years old, decreased SE, and elevated total cholesterol (TC). In univariate analysis, JS correlated significantly with a pre-AI % YAM of ≥80%, decreased SE, and elevated TC. Eight (7.7%) patients maintained SE at ≥5 pg/mL for >6 consecutive months, with no JS. In chi-square testing, hot flashes showed a significantly higher incidence in patients <55 years old. *Conclusion*. AI-ADRs occurred more readily in younger patients. Decreased SE may be indirectly involved in JS.

## 1. Background

An aromatase inhibitor (AI) showed efficacy that was superior to that of tamoxifen (TAM) in large-scale randomized clinical trials in breast cancer patients, and AIs are now the most extensively used drugs for postoperative adjuvant therapy for hormone-dependent postmenopausal breast cancer [1, 2]. However, on the minus side, manifestation of joint symptoms (joint pain and stiffness) as drug-related adverse reactions of AIs has become an important safety issue. Joint symptoms have been variously reported to occur at an incidence of 20% to 36% in patients administered AIs, and discontinuation of administration of AIs is sometimes unavoidable [3, 4]. It has been said that a decrease in serum estrogen (E2) is associated with the causation of joint symptoms due to AI administration [5–10]. However, there have been no reports of stringent studies of such an association, and the detailed mechanisms involved in any such association thus remain unclear.

In recent years, there have been reports that AIs are poorly metabolized in some patients [11], while in other patients AIs do not show efficacy because of genomic polymorphism of the metabolizing enzymes [12]. Since March of 2008 we have been monitoring the E2 levels in breast cancer patients who were started on AI therapy, and we reported that some patients show renewed elevation of E2 in spite of being thought to be clinically postmenopausal [13].

The present prospective study was designed with the objective of elucidating whether or not there truly is an association between manifestation of joint symptoms in response to AI administration and a decrease in serum E2. Accordingly, patients who were administered an AI were monitored with regard to drug-related adverse reactions, including joint symptoms, their clinical findings, and the E2 level. Statistical testing was performed to identify possible significant correlations.

## 2. Patients and Methods

A total of 159 hormone-dependent postmenopausal breast cancer patients were started on administration of an AI at Tokushima Breast Care Clinic during the period from March 2008 through October 2009. The AI treatment consisted of anastrozole, in an oral dose of 1 mg/day. Menopause was defined as the state of having undergone bilateral oophorectomy, age of 60 years or older, or an age of under 60 years with amenorrhea for at least 12 months, and serum E2, LH, and FSH levels that satisfy the diagnostic criteria for postmenopause. For each enrolled patient, the following information was elucidated and recorded: the patient's age at enrollment, age at menarche, number of childbirths, BMI, age at menopause, time interval from menopause until AI administration, presence/absence of therapy prior to AI administration, and clinicopathological findings. In addition, one adverse reaction of AI inhibitors is elevation of serum cholesterol, a lipid metabolism abnormality [14]. For this reason, we carried out measurement of both E2 and total cholesterol in this study. The serum levels of total cholesterol (TC) and E2 were assayed prior to administration of the AI and then at 3, 6, 9, and 12 months after starting the AI therapy. The TC assays were performed by FALCO Biosystems Ltd. (Kyoto, Japan), and the serum E2 level was measured by ECLIA (electrochemiluminescence immunoassay) in this laboratory. The laboratory's standard values for the postmenopausal levels of those hormones are 130–219 mg/dL for TC and 10–40 pg/mL for E2 (lower limit of detection: 5 pg/mL). Patients who maintained E2 at ≥5 pg/mL for more than 6 consecutive months following the start of AI administration were classified as “rebound” cases. All other patients were classified as “decreased” cases. Patients who showed a transient increase or did not show an initial decrease in E2 after the start of AI administration but who then showed a decrease within 6 months following AI administration were defined as “decreased” cases.

Similarly, patients whose serum TC level was 10 IU/mL or more for at least 6 months following the start of AI administration were classified as “elevated” cases, while all other patients were classified as “nonelevated” cases. In addition, the bone density was determined prior to the start of AI administration and then every 4 months thereafter. At each scheduled time of bone density testing the patients were asked whether they experienced drug-related adverse reactions, that is, joint symptoms and hot flashes, which are known to occur at high incidences with AIs. The bone density was measured on the basis of the speed of sound (SOS) for propagation of an ultrasound pulse through the calcaneal bone. The bone density was expressed as the percent of the SOS relative to that of the young adult mean value (%YAM) and the percent of the SOS relative to that of the age-matched mean value (%AGE). Patients showing a decrease in the bone density of 10% or more that persisted for at least 4 months were classified as “decreased” cases, while all other patients were classified as “nondecreased” cases. Drug-related adverse reactions were evaluated in accordance with the Common Terminology Criteria for Adverse Events (CTCAEs) Version 4.0 [15].

The analyzed population of this study consisted of 104 patients who had not experienced joint symptoms or hot flashes prior to AI administration and were observed and subjected to the above clinical testing for at least 6 months subsequent to the start of AI administration. In addition, patients who had ingested a concomitant medication that could be thought to be related to an adverse reaction were excluded from the analysis of that drug-related adverse reaction.

Statistical analyses were performed using the chi-square test and trend analysis by logistics regression analysis. A *P* value of <.05 was defined as representing a significant difference.

The design of this study was approved by the Ethics Committee of The Institute of Medical Science, The University of Tokyo, and The University of Tokushima (Protocol no.: 19-11-1211). Prior informed consent was obtained in writing from each of the enrolled patients.

## 3. Results

It is necessary to acknowledge that a limitation of this study is that the study population was small. The clinicopathological characteristics of the 104 analyzed patients were as follows. The disease stage was Stage 0 in 11 patients, Stage I in 58 patients, and Stage II in 35 patients, and 25 patients had axillary lymph node metastases. Sixty-six patients had not received any treatment prior to AI administration, while 21 patients had been administered TAM monotherapy and 10 patients had received chemotherapy alone (Table 1).

The mean follow-up period for the 104 patients was 14.6 months, and 8 (7.7%) of the patients were classified as E2 rebound cases. In this study, patients who showed continuous E2 elevation even after the start of AI administration and patients who showed no decrease in E2 after the start of AI administration were classified as “rebound” cases. However, because the sample size of this study was a bit small, these two types of patients were pooled and analyzed as “rebound” cases. In addition, because the lower limit of detection for E2 was 5 pg/mL, it was impossible to identify patients whose E2 level did not decrease among the patients (32/104 patients; 30.8%) with a baseline E2 level below that lower limit of detection. 

The incidences of AI-related adverse reactions were 31.7% (33 patients) for joint symptoms and 18.3% (19 patients) for hot flashes. The severity of the joint symptoms was Grade 1 in 22 patients, Grade 2 in 7 patients, and Grade 3 in 4 patients.

Chi-square testing for possible correlations between joint symptoms and various background factors found that the incidence of joint symptoms was significantly higher in the patient group aged less than 55 years (*P* < .05). In addition, the patient group with less than a 5-year interval between menopause and the start of AI therapy showed a tendency to experience more joint symptoms than the patient group with an interval of 10 or more years (*P* = .056). Moreover, none of the patients in the E2 rebound patient group experienced joint symptoms, and the incidence of joint symptoms was significantly lower than in the E2 decreased patient group (*P* < .05). The TC-elevated patient group showed a significantly higher incidence of joint symptoms compared with the TC-nonelevated patient group (*P* < .05). In univariate analysis, joint symptoms were found to manifest at a significantly higher incidence in the following patient strata: a preAI %YAM of 80% or more (*P* < .05), E2 decreased (*P* < .05), and TC elevated (*P* < .01). The time interval between menopause and the start of AI therapy showed a tendency to influence the manifestation of joint symptoms (*P* = .059). Multivariate analysis did not find any significant correlations between the manifestation of joint symptoms and the analyzed patient factors (Table 2).

Chi-square testing and univariate analysis for possible correlations between hot flashes and various background factors found that the incidence of hot flashes was significantly higher in the following patient strata: age of less than 55 years (*P* < .05, *P* < .05), menarche at an age of less than 12 years (*P* < .05, *P* < .05), no prior therapy (chisquare testing only: *P* < .01), less than a 5-year interval between menopause and the start of AI therapy (*P* < .01, *P* < .01), a preAI %YAM of 80% or more (*P* < .05, *P* < .01), and TC elevated (*P* < .01, *P* < .05). Multivariate analysis did not find any significant correlations between the manifestation of hot flashes and the analyzed patient factors (Table 3).

## 4. Discussion

Aromatase inhibitors (AIs) are currently the drugs of choice for postoperative adjuvant therapy for hormone-dependent postmenopausal breast cancer [1, 2]. However, manifestation of joint symptoms (joint pain and stiffness) as drug-related adverse reactions of AIs has become an important safety issue [3, 4]. A decrease in E2 has been said to be one cause of the joint symptoms associated with AI administration [5–10]. However, the possible mechanism of such causation remains unclear, and there have been no stringent studies of such an association between a decrease in E2 and manifestation of joint symptoms. Accordingly, with the aim of elucidating the detailed mechanisms involved in AI-related adverse reactions, the present study was designed and executed by prospectively surveying adverse reactions and measuring the E2 level in postoperative postmenopausal breast cancer patients who were administered an AI (anastrozole).

Joint symptoms manifested at an incidence of 31.7% in these AI-treated patients, and they interfered with the activities of daily life of approximately 4% of the patients. It has been said that joint symptoms in AI-treated patients tend to occur more readily if the interval between menopause and the start of AI therapy is within 5 years and if the patient's age is 50–59 years [16]. Our present study results also show that joint symptoms tended to occur at a higher incidence in younger patients, when the interval between menopause and the start of AI therapy was shorter and when the pre-AI %YAM was higher. It can thus be surmised that factors associated with younger patients are more likely to lead to manifestation of joint symptoms. Other factors that have been suggested to be involved in the manifestation of joint symptoms include the presence of prior therapy and obesity, but the published literature does not show any consensus in this regard [3, 5].

We also analyzed the manifestation of hot flashes in our study population. The incidence of hot flashes in this cohort was 18.3%, which was lower than the incidence of joint symptoms. However, hot flashes also tended to manifest more frequently in the same younger patient population as joint symptoms. TC elevation was associated with both joint symptoms and hot flashes, but a decrease in bone density did not show any association with either adverse reaction. These differences might be attributable to the fact that TC elevation is a change that manifests in a short time-frame, whereas a decrease in bone density is a change that occurs over a longer period of time.

The E2 level was also analyzed for possible association with the manifestation of AI-related adverse reactions. Our results showed that hot flashes were not associated with the E2 level. Hot flashes are considered to be a functional change that occurs under sympathetic nerve stimulation due to a decrease in E2, but it was reported that they do not correlate with the E2 value [17]. Thus, it has been surmised that hot flashes do not occur as a result of a simple decrease in E2.

On the other hand, with regard to the possibility of association of joint symptoms with E2, joint symptoms did not occur in patients with E2 rebound, whereas their manifestation was associated with a decrease in E2. However, multivariate analysis did not show a clear significant difference in relation to a decrease in E2. Accordingly, the results indicated the possibility that a decrease in E2 is not directly associated with manifestation of joint symptoms and instead suggested the possibility that a decrease in E2 causes some other secondary change(s) in joints, which is followed by manifestation of joint symptoms.

Aromatase is present in osteoblasts, synovial cells, and chondrocytes of articular cartilage, and one report surmised that a local decrease in the E2 level in bones and joints is involved in joint symptoms [4]. Some studies used sonography and magnetic resonance imaging to objectively investigate the local findings in patients with joint symptoms due to AI therapy [18, 19]. It was reported that carpal tunnel syndrome, wrist effusion, tendon sheath enhancement and thickening, and tenosynovial changes were seen in patients with joint symptoms due to AI therapy, findings that suggest the possibility that joint symptoms are caused by secondary organic changes that occur in joints.

For the present study, we used the ECLIA method to measure the E2 levels; this method has a lower limit of detection of 5 pg/mL. This level of sensitivity can be considered a drawback of this study, since some of the enrolled patients had an E2 level which was too low to detect even before the start of AI administration. Stanczyk et al. reported on the disadvantages of various E2 assay techniques [20]. A liquid chromatography-tandem mass spectrometry (LC-MS/MS) method has become available in recent years, which permits more sensitive assay of E2 [11, 21]. In the future, it will be necessary to prospectively investigate organic changes in joints during AI therapy by using this highly sensitive assay technique to monitor E2 levels and also applying imaging modalities. An additional drawback of our current study is the small number of analyzed cases, and there is a need to perform a future study in a larger cohort.

## 5. Conclusion

A limitation of this study is the fact that the study population was small. AI-related adverse reactions occurred more readily in younger patients, and the chi-square test and univariate analysis both showed that a decrease in E2 was associated with manifestation of joint symptoms. These results indicate that joint symptoms due to AI therapy are affected by a decrease in E2. However, multivariate analysis did not show any clear association of a decrease in E2 with joint symptoms, suggesting the possibility that a decrease in E2 is indirectly involved in the manifestation of those symptoms.

##  Competing Interests 

The authors declare that they have no competing interests.

## Figures and Tables

**Table 1:** The Clinicopathological characteristics (*n* = 104).

Stage	
0	11
I	58
II	35
Nodal status	
*n* (−)	79
*n* (+)	25
Therapy prior to AI administration	
None	66
TAM	21
CT	10
TAM+LH-RH analogue	2
CT+TAM	4
CT+TAM+LH-RH analogue	1

**Table 2:** Clinical parameters of patients with joint symptoms and without joint symptoms (*n* = 104).

		With joint Symptoms *n* = 33	Without joint symptoms *n* = 71	*P* value of chi square tests	Univariate Analysis^a^		Multivariate Analysis^a^	
					Odds ratio (95% CI)	*P* value	Odds ratio (95% CI)	*P* value
Age (years)	<55 55–65 65≤	5 19 9	3 34 34	*P* < .05	0.43 (0.21–0.86)	<.05	0.47 (0.21–1.05)	NS
Age at menarche (years)	<12 12–15 15≤	2 30 1	3 57 11	NS	0.37 (0.11–1.21)	NS		
No. of childbirths	None 1-2 2≤	2 24 7	8 21 42	NS	1.14 (0.54–2.41)	NS		
BMI (kg/m^2^)	<25 25–30 30≤	18 13 2	44 21 6	NS	1.13 (0.60–2.15)	NS		
Therapy prior to AI administration	None Yes	18 15	48 23	NS	Not analyzed^b^			
Time from menopause^c^ (years)	<5 5–10 10≤	8 11 14	8 21 42	NS *P* = .056 (<5 ver 10≤)	0.59 (0.34–1.02)	.059		
Pre-AI administration %YAM (%)	<70 70–80 80≤	6 12 12	25 30 12	NS	1.98 (1.09–3.61)	<.05	1.65 (0.84 – 3.21)	NS
Pre-AI administration %AGE (%)	<80 80–100 100≤	4 11 15	10 31 27	NS	1.28 (0.68–2.39)	NS		
Change of E2	Rebound Decreased	0 33	8 63	*P* < .05	2.52 (1.01–6.26)	<.05	2.28 (0.70 – 7.40)	NS
Change of T-chol^d^	Elevated Non-elevated	16 13	15 49	*P* < .05	0.30 (0.13–0.71)	<.01	0.52 (0.22 – 1.25)	NS
Change of %AGE^d^	Not -decreased Decreased	17 13	44 22	NS	1.63 (0.70–3.77)	NS		

BMI: body mass index; AI: aromatase inhibitor; %YAM: % young adult mean value; %AGE: % age-matched mean value; E2: estradiol; T-chol: total cholesterol; NS: not significance. ^a^Maltivariate logistic regression analysis (trend analysis). ^b^Because of the diversity of the treatment methods employed, statistical analyses were not performed. ^c^Time from menopause until AI administration. ^d^Excluded patients with medicine.

**Table 3:** Clinical parameters of patients with hot flashes and without hot flashes (*n* = 104).

		With joint Symptoms *n* = 33	Without joint symptoms *n* = 71	*P* value of chi square tests	Univariate Analysis^a^		Multivariate Analysis^a^	
					Odds ratio (95% CI)	*P* value	Odds ratio (95% CI)	*P* value
Age (years)	<55 55–65 65≤	4 11 4	4 42 39	*P* < .05	0.33 (0.14–0.78)	<.05	0.81 (0.18–3.63)	NS
Age at menarche (years)	<12 12–15 15≤	3 15 1	2 72 11	*P* < .05	0.21 (0.05–0.95)	<.05	0.48 (0.07–3.27)	NS
No. of childbirths	None 1-2 2≤	2 12 5	8 59 18		1.14 (0.46–2.83)	NS		
BMI (kg/m^2^)	<25 25–30 30≤	8 10 1	54 24 7		1.53 (0.73–3.21)	NS		
Therapy prior to AI administration	None Yes	7 12	59 26	*P* < .01	Not analyzed^b^			
Time from menopause^c^ (years)	<5 5–10 10≤	7 8 4	9 24 52	*P* < .01	0.31 (0.16–0.63)	<.01	0.42 (0.13–1.37)	NS
Pre-AI administration %YAM (%)	<70 70–80 80≤	1 8 8	30 34 17	*P* < .05	2.98 (1.35–6.59)	<.01	2.63 (0.99–7.01)	.053
Pre-AI administration %AGE (%)	<80 80–100 100≤	2 6 9	12 36 33		1.38 (1.35–3.03)	NS		
Change of E2	Rebound Decreased	1 18	7 78		1.62 (0.19–13.9)	NS		
Change of T-chol^d^	Elevated Non-elevated	10 6	21 56	*P* < .01	0.31 (0.11–0.88)	<.05	0.43 (0.13–1.43)	NS
Change of %AGE^d^	Not -decreased Decreased	10 7	51 28		1.36 (0.50–3.73)	NS		

BMI: body mass index; AI: aromatase inhibitor; %YAM: % young adult mean value; %AGE: % age-matched mean value; E2: estradiol; T-chol: total cholesterol; NS: not significance. ^a^Maltivariate logistic regression analysis (trend analysis). ^b^Because of the diversity of the treatment methods employed, statistical analyses were not performed. ^c^Time from menopause until AI administration. ^d^Excluded patients with medicine.
